# Lactation-related metabolic mechanism investigated based on mammary gland metabolomics and 4 biofluids’ metabolomics relationships in dairy cows

**DOI:** 10.1186/s12864-017-4314-1

**Published:** 2017-12-02

**Authors:** Hui-Zeng Sun, Kai Shi, Xue-Hui Wu, Ming-Yuan Xue, Zi-Hai Wei, Jian-Xin Liu, Hong-Yun Liu

**Affiliations:** Institute of Dairy Science, MoE Key Laboratory of Molecular Animal Nutrition, College of Animal Sciences, Zhejiang University, Hangzhou, 310058 People’s Republic of China

**Keywords:** Biofluids relationship, Dairy cow, Lactation, Mammary gland, Metabolic function, Metabolomics

## Abstract

**Background:**

Lactation is extremely important for dairy cows; however, the understanding of the underlying metabolic mechanisms is very limited. This study was conducted to investigate the inherent metabolic patterns during lactation using the overall biofluid metabolomics and the metabolic differences from non-lactation periods, as determined using partial tissue-metabolomics. We analyzed the metabolomic profiles of four biofluids (rumen fluid, serum, milk and urine) and their relationships in six mid-lactation Holstein cows and compared their mammary gland (MG) metabolomic profiles with those of six non-lactating cows by using gas chromatography-time of flight/mass spectrometry.

**Results:**

In total, 33 metabolites were shared among the four biofluids, and 274 metabolites were identified in the MG tissues. The sub-clusters of the hierarchical clustering analysis revealed that the rumen fluid and serum metabolomics profiles were grouped together and highly correlated but were separate from those for milk. Urine had the most different profile compared to the other three biofluids. Creatine was identified as the most different metabolite among the four biofluids (VIP = 1.537). Five metabolic pathways, including gluconeogenesis, pyruvate metabolism, the tricarboxylic acid cycle (TCA cycle), glycerolipid metabolism, and aspartate metabolism, showed the most functional enrichment among the four biofluids (false discovery rate < 0.05, fold enrichment >2). Clear discriminations were observed in the MG metabolomics profiles between the lactating and non-lactating cows, with 54 metabolites having a significantly higher abundance (*P* < 0.05, VIP > 1) in the lactation group. Lactobionic acid, citric acid, orotic acid and oxamide were extracted by the S-plot as potential biomarkers of the metabolic difference between lactation and non-lactation. The TCA cycle, glyoxylate and dicarboxylate metabolism, glutamate metabolism and glycine metabolism were determined to be pathways that were significantly impacted (*P* < 0.01, impact value >0.1) in the lactation group. Among them, the TCA cycle was the most up-regulated pathway (*P* < 0.0001), with 7 of the 10 related metabolites increased in the MG tissues of the lactating cows.

**Conclusions:**

The overall biofluid and MG tissue metabolic mechanisms in the lactating cows were interpreted in this study. Our findings are the first to provide an integrated insight and a better understanding of the metabolic mechanism of lactation, which is beneficial for developing regulated strategies to improve the metabolic status of lactating dairy cows.

**Electronic supplementary material:**

The online version of this article (10.1186/s12864-017-4314-1) contains supplementary material, which is available to authorized users.

## Background

Dairy cows contribute the most important source of dairy foods for humans, and this relies largely on the cows’ lactation performance [1]. The inherent metabolic status during lactation and the difference from the non-lactation period are the most important metabolic biological processes for determining milk production and sustainability[2], and they depend on the development of the mammary gland (MG) and the overall coordinated metabolism and physiology.

Metabolomics, a vital part of systemic biology, can be utilized to explore the disease, diet or environment-related comprehensive metabolism by analyzing endogenous small molecules in the biofluids or tissues [3]. To date, biofluid metabolomics combined with tissue metabolomics has provided valuable information on the overall or partial metabolic mechanisms [4]. Biofluid metabolomics has been extensively applied in disease diagnosis [5], biomarker discovery [6] and novel pathway identification [7] in human clinical studies. Recently, biofluid metabolomics has been widely used to investigate the relationship between the rumen fluid metabolites and cow health [8], the effect of heat stress on milk metabolites [9], and the urinary biomarkers under different quality forage diets [6]. However, most biofluid metabolomics studies in dairy cows have been limited to a single biofluid and/or focused on the changes in the physiological conditions under different treatments. Multiple biofluid metabolomics and their relationships under the same treatment allow new insights into the inherent metabolic pattern and global metabolism, which could contribute to improving the metabolic status for better productivity and sustainability. Tissue metabolomics can capture the subtle metabolic variation of the specific organs or parts of the body and reflect the actual biological processes that affect gene expression, transcription and translation [10]. It has been revealed that MG metabolomics is an effective way to explore the initiation, maintenance and regulation of lactation [11, 12] and can provide direct connections with the milking phenotypes. Therefore, identifying the metabolites in the MG during lactation and comparing their key pathways with non-lactating cows could enhance our understanding of the lactation mechanism.

In this study, we hypothesize about the inherent metabolic characteristics during lactation and their differences from the non-lactation period, which can be addressed by biofluid metabolomics combined with tissue metabolomics. The relationships among four biofluids’ (rumen fluid, serum, milk and urine) metabolomics profiles from the lactating cows were analyzed to investigate the inherent metabolic patterns and possible effects on lactation sustainability. The MG metabolomics profiles were compared between lactating and non-lactating cows to identify the key metabolites and pathways and their potential regulatory roles in lactation.

## Methods

The experimental procedures were approved by the Animal Care Committee at Zhejiang University (Hangzhou, China) and were in accordance with the university’s guidelines for animal research.

### Study design and sample collection

In total, 12 Holstein dairy cows, 6 lactating and 6 non-lactating, were used in this study. Among them, 6 multiparous dairy cows with similar milk yields (30.4 ± 2.29 kg/d, mean ± SD) and at similar lactation stages (days in milk = 164 ± 19.6 d, mean ± SD) were fed a diet with a forage-to-concentrate ratio of 45:55, and they were provided 16.7% (DM basis) crude protein and 1.57 Mcal/kg net energy for lactation, following the protocol described in our previous study [13]. The diet ingredients and nutrient composition are commonly applied in state-of-the-art farms throughout China (Additional file 1: Table S1). Approximately 1000 mg of MG tissues was collected immediately after slaughter according to previously described methods [14] from each cow in both the lactation and non-lactation groups. The tissues were then stored at −80 °C until metabolite extraction. Biofluid samples, including 50 mL of rumen fluid, 50 mL of milk, 10 mL of blood and 10 mL of urine, were collected from the lactating cows before the morning feeding at 6:00 AM. Rumen fluid was collected using an oral stomach tube following the standard procedures [15], milk samples were collected using a milk sampling device (Waikato Milking Systems NZ Ltd., Waikato, Hamilton, New Zealand), blood samples were collected from the jugular vein using pro-coagulation 10-mL tubes, and urine samples were collected using the vulval stimulation method. To minimize any possible degradation of the metabolites, rumen fluid, milk and urine samples were infused into a 15-mL sterilized centrifuge tube and immediately placed in liquid nitrogen. Before storage in a − 80 °C freezer, the rumen fluid, milk and urine samples were centrifuged at 6000×g, 3000×g, and 3000×g, respectively, and incubated at 4 °C for 15 min. Blood samples were centrifuged at 4 °C, 3000×g for 15 min within 20 min after sample collection. More details can be obtained from our previous study [16].

### Extraction of compounds

The biofluid metabolomics was examined using an Agilent 7890 gas chromatography system equipped with a Pegasus 4D time of flight mass spectrometer (LECO, St. Joseph, MI, USA) [6]. The tissue metabolomics procedures were performed as follows. First, 100 mg of MG tissue from each sample was added to a 2-mL Eppendorf tube with 0.4 mL of methanol-chloroform (V_methanol:_ V_chloroform_ = 3:1) and 30 μL of L-2-chlorophenylalanine (1 mg/mL, stored in dH_2_O) and was mixed by vortexing for 10 s. Second, steel balls were placed in the tube and milled for 5 min at 55 Hz. The sample was then centrifuged at 4 °C at 12,000 rpm for 15 min. Third, approximately 0.4 mL of supernatant was transferred into a 2 mL silylated vial. An equal volume (10 μL) of each sample was placed in a new 2 mL silylated vial as a mixed sample for the quality control of the stability of the equipment system, the standard deviation of the beginning, middle and ending retention time of the mixed samples was less than 0.2, which indicates good stability.

### Metabolite derivatization

The extracts were dried in a vacuum concentrator at 30 °C for 1.5 h. Methoxymethyl amine salt (80 μL; dissolved in pyridine to a final concentration of 20 mg/mL) was added to the dried metabolites, mixed and gently sealed. The solution was then incubated at 37 °C for 2 h in an oven. Then, 100 μL of bis trifluoroacetamide (containing 1% trimethylchlorosilane, *v*/v) was added to each sample, which was sealed again and incubated at 70 °C for 1 h. In addition, 10 μL of fatty acid methyl esters (a standard mixture of fatty acid methyl esters, C8-C16: 1 mg/mL; C18-C30: 0.5 mg/mL in chloroform) was added to the mixed sample and cooled to room temperature. Finally, the samples were mixed well and subjected to gas chromatography-time of flight/mass spectrometry (GC-TOF/MS) testing.

### Metabolite identification by GC-TOF/MS

GC-TOF/MS analysis of the tissue samples was performed on an Agilent 7890 gas chromatograph system in cooperation with a Pegasus HT time-of-flight mass spectrometer (LECO, St. Joseph, MI, USA). The system used a DB-5 MS capillary column coated with 5% cross-linked diphenyl and 95% dimethyl polysiloxane (30 m × 250 μm inner diameter, 0.25 μm film thickness; J&W Scientific, Folsom, CA, USA). One μL aliquot of the analyte was added in splitless mode. Helium was used as the carrier gas. The flow of the front inlet purge was 3 mL min^−1^, and the gas flow rate through the column was 20 mL min^−1^. The original temperature was set at 50 °C and was maintained for 1 min. The temperature was increased to 330 °C at a speed of 10 °C min^−1^ and was maintained for 5 min at 330 °C. Temperatures of 280 °C, 280 °C, and 220 °C were used for the injection, transfer line, and ion source, respectively. The energy was −70 eV in electron influence mode. The full-scan mode of the mass spectrometry data was 85 m/z - 600 m/z at a rate of 20 spectra per second after a solvent delay of 366 s.

### Identification of differentially expressed metabolites

Using the interquartile range denoising method, missing values of the raw data were filled by half of the minimum value and valid peaks were detected and only the metabolites remained. Additionally, an internal standard (L-2-chlorobenzene alanine) log normalization method was applied. The resulting three-dimensional data, including the peak number, sample name and normalized peak area, were uploaded to the online analysis platform - Metaboanalyst 3.0 (http://www.metaboanalyst.ca/) for univariate and multivariate analyses. Both one-way ANOVA and post hoc analysis were used to identify the important metabolites of the four biofluids. When the *P* value was less than 0.05, the metabolite was characterized as differentially expressed. Univariate analyses provided a preliminary overview of the features that were potentially significant. The false discovery rate (FDR) was used to conduct multiple comparisons testing, with an FDR value less than 0.05 indicating significance. Multivariate analyses included hierarchical cluster analysis (HCA), principal component analysis (PCA), partial least squares discriminant analysis (PLS-DA), and orthogonal projections to latent structures (OPLS). PCA was used to visualize the dataset of multitudinous variables in a 2 or 3-dimensional figure and display the general similarity and difference. The first principal component of the PLS (variable importance projection, VIP value) was obtained to identify the differentially expressed metabolites. Variables with VI*P* values exceeding 1.0 and *P* values less than 0.05 were selected as differentially expressed metabolites. An S-plot was generated to further identify the statistically significant and potentially biochemically significant metabolites in both their contributions to the module variables and the reliability of the module.

### Pathway characterization

The compound names and relative concentrations of the shared metabolites were imported into Metaboanalyst 3.0 (http://www.metaboanalyst.ca) to perform functional enrichment and impact pathway analyses (integrates pathway enrichment analysis and pathway topology analysis). The functional enrichment analysis was based on several libraries containing approximately 6300 groups of biologically meaningful metabolite sets. Functional enrichment analysis was performed using the metabolite set enrichment analysis (MSEA) to investigate the enrichment of the predefined groups of functionally related metabolites, which are usually associated with biological pathways [17]. Significant functional pathways were selected using a fold enrichment (FE) threshold greater than 2.0 and an FDR value less than 0.05 [18]. Pathway topological analysis was used to calculate the importance of the metabolites based on the out-of-degree centrality and the relative betweenness measures of a metabolite in a given metabolic network [19]. The impact pathway value was calculated as the sum of the matched important metabolites of all the metabolites in each pathway [20]. Impact values greater than 0.10 and *P* values less than 0.05 were defined as significant impact pathways. Extremely different pathways were defined with *P* values less than 0.01. Additionally, commercial databases, including the Kyoto Encyclopedia of Genes and Genomes (KEGG, http://www.genome.jp/kegg/) and the Small Molecular Pathway Database (SMPDB, http://smpdb.ca), were used to search for metabolites and for the integrated pathway analysis.

## Results

### Lactation performance

The 4% fat-corrected milk yield of the six lactating cows was 26.29 ± 2.66 kg/d (mean ± SD), and the milk protein, milk fat and lactose contents were 3.29 ± 0.16%, 4.17 ± 0.37% and 4.91 ± 0.14% (mean ± SD), respectively. Detailed results can be found in our previous study [21]. No adverse events occurred in any animal.

### Metabolites identified in biofluids using GC-TOF/MS

From the metabolomics datasets of the four biofluids in six lactating cows, 33 shared metabolites were identified in the rumen fluid, serum, milk and urine, including noradrenaline, methylmalonic acid, glycine, lyxose, L-malic acid, thymol, creatine, 5-methoxytryptamine, oxoproline, glycerol, L-threose, m-cresol, aminomalonic acid, 2-hydroxybutanoic acid, alanine, hydroxylamine, phosphate, fumaric acid, glucose, prostaglandin E2, isoleucine, lactose, malonic acid, N-methyl-L-glutamic acid, phenylethylamine, 2,4-diaminobutyric acid, oxalic acid, lactic acid, 4-androsten-1-beta-ol-3,17-dione, norleucine, asparagine, conduritol-b-epoxide, and 5-aminovaleric acid. The relative abundance of each metabolite is presented in Additional file 1: Table S2.

**Fig. 1:**
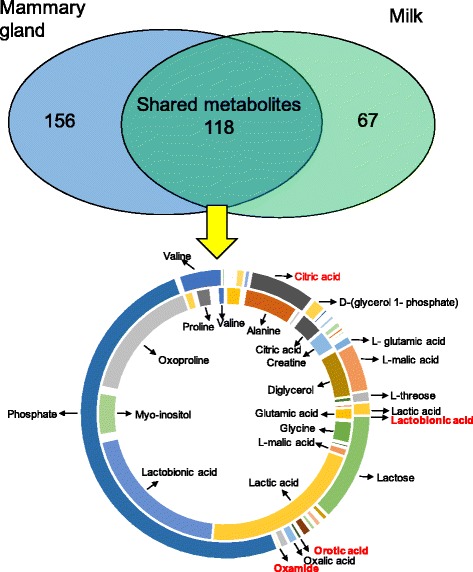
Metabolite comparison profiling of mammary gland tissue and milk from the same 6 lactating dairy cows. In total, 118 shared metabolites, 156 mammary gland-specific metabolites and 67 milk-specific metabolites were identified. The 118 shared metabolites from the mammary gland tissue (inside) and milk (outside) are displayed in the ring chart. The potential biomarkers in the mammary gland metabolomics study were also identified in milk and are marked in red

When we compared the MG metabolomics and milk metabolomics of the 6 same lactating dairy cows in the lactation group, a total of 118 shared metabolites, 156 MG-specific metabolites and 67 milk-specific metabolites were identified (Fig. 1, Additional file 1: Table S3).

### Metabolites identified in the MG tissue using GC-TOF/MS

In total, 394 valid peaks were detected from the analytes of the MG tissues. Based on the LECO/Fiehn Metabolomics Library, 274 metabolites were characterized and quantified (Additional file 1: Table S4). Among them, stigmasterol, sarcosine, palatinitol, oxamide, lyxose, L-threose, D-erythronolactone, cholesterol, carnitine and carbamoyl-aspartic acid were identified in the lactation group only and mainly belong to lipid or lipid-like molecules, carbohydrates and amino acids. Conversely, terephthalic acid, phenyl beta-D-glucopyranoside, octadecanol and alpha-ketoisocaproic acid were found only in the non-lactation dairy cow group.

### Multivariate statistical analysis

**Fig. 2:**
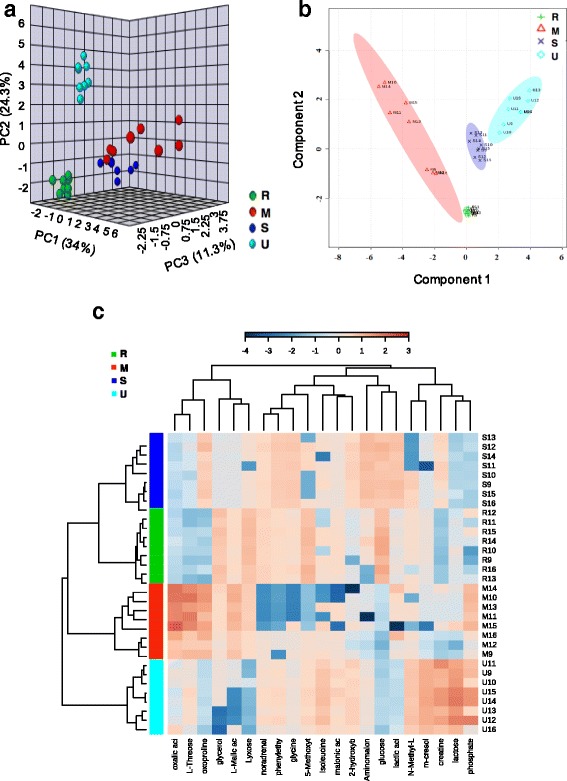
The 3-D principal component analysis (PCA) score map, 2-D partial least squares discriminant analysis (PLS-DA) score map, and hierarchical clustering analysis (HCA) for the shared metabolites in the rumen fluid, serum, milk and urine derived from gas chromatography-time of flight/mass spectrometry. Subgraph **a**, **b** and **c** represent the PCA, PLS-DA and HCA, respectively. The green, dark blue, red and light blue represent the samples from the rumen fluid, serum, milk and urine, respectively. The patterns shown in each row were categorized using an average linkage hierarchical clustering program. The light blue boxes indicate an expression ratio that is less than the mean, and dark red boxes denote an expression ratio that is greater than the mean. The tree clusters and their shorter Euclidean distances indicate higher similarities. The similarity between the two metabolites is represented by the branch height; the lower a node is vertically, the more similar its subtree is

The 3D–PCA score plot of the metabolic profiles showed significantly separated clusters among the four biofluids (Fig. 2a). All score plots for the rumen fluid, serum, milk, and urine sample were in the Hotelling T^2^ ellipse with 95% confidence. As shown in the PLS-DA score map, the samples were clearly separated into four parts, indicating the differential metabolic profiling of the four biofluids samples (Fig. 2b). HCA revealed sub-clusters containing varying numbers of metabolites, with different metabolomics profiles from each biofluid being clustered together (Fig. 2c). The metabolites from the rumen and serum samples were clustered together, which then clustered with the milk samples, but the cluster derived from the urine samples was separated from the other three biofluids. Several metabolites with higher relative concentrations were identified in each biofluid: glycerol, noradrenaline, lyxose, 5-methoxytryptamine and glucose in rumen fluid; lactic acid, glycine, N-methyl-L-glutamic acid, phenylethylamine, aminomalonic acid and glucose in the serum; glycerol, oxoproline, oxalic acid, L-malic acid, lyxose and L-threose in the milk; and isoleucine, m-cresol, 2-hydroxybutanoic acid, phosphate, creatine and lactose in the urine. HCA also revealed the presence of one cluster that included an amino acid and its derivatives (e.g., glycine, aminomalonic acid, N-methyl-L-glutamic acid, 5-methoxytryptamine, noradrenaline and phenylethylamine) and an organic acid and its derivatives (e.g., glucose, lactic acid and malonic acid). Another cluster consisted of aliphatic compounds, such as oxoproline, L-threose, oxalic acid, L-malic acid, glycerol and lyxose.

**Fig. 3:**
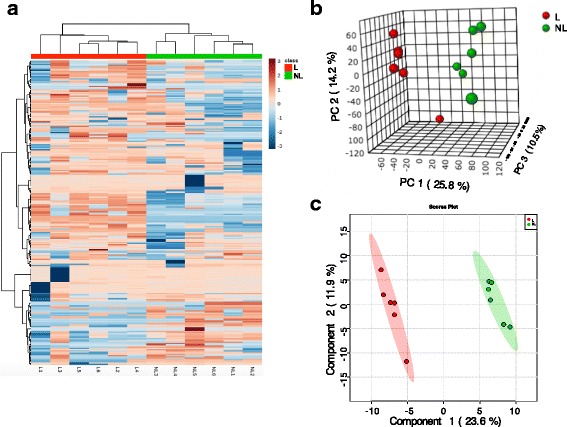
Heatmap, 3-D principal component analysis (PCA) score map and 2-D partial least squares discriminant analysis (PLS-DA) score map of the mammary gland tissue from 6 lactating cows and 6 non-lactating cows. Subgraph **a**, **b** and **c** represent the Heatmap, PCA, and PLS-DA, respectively. Red and green color bars represent lactating and non-lactating cows, respectively. The blue boxes indicate an expression ratio that is less than the mean, and the red boxes denote an expression ratio that is greater than the mean. The darker the color is, the larger the difference there is from the mean value

For the MG metabolomics, the heat map revealed the 6 lactating and 6 non-lactating dairy cows were assigned to 2 significantly separated clusters (Fig. 3a). The overview of the heatmap showed that the concentrations of most MG metabolites in the lactation group were higher than those in MG of non-lactating dairy cows. The 3-D PCA score map of GC-TOF/MS metabolic profiles of MG tissue showed significant discrimination between the two animal groups (Fig. 3b). All score plots for the MG samples in the 2 groups were in the 95% Hotelling T^2^ ellipse in the PLS-DA score map (Fig. 3c). In summary, clear separation in metabolomic profiles was found between the lactating and non-lactating dairy cow groups.

### Identification of differentially expressed metabolites

**Fig. 4:**
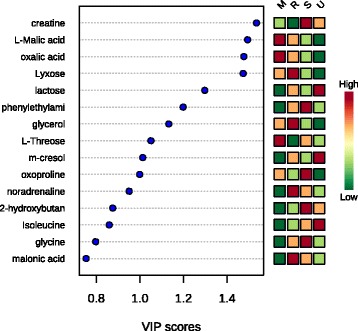
Differentially expressed metabolites in the rumen fluid, serum, milk and urine of the lactating dairy cows identified by partial least squares discriminant analysis (PLS). The colored boxes on the right indicate the relative concentrations of the corresponding metabolite in each biofluid. R: rumen fluid; M: milk; S: serum; U: urine. Darker green indicates a lower relative concentration and darker red represents a higher relative concentration. The x-axis represents the first principal component of the PLS (variable importance projection, VIP value), and the blue dots represent the different metabolites

The differentially expressed metabolites identified from the four biofluids are listed in Additional file 1: Table S5. Twenty-six metabolites showed obvious differences among the four biofluids, with four shared metabolites being differentially expressed in each comparison (FDR value <0.01): oxoproline, L-threose, creatine and lactose. As illustrated in Fig. 4, the VIP values of the PLS-DA model >1 included creatine, L-malic acid, oxalic acid, lyxose, lactose, phenylethylamine, glycerol, L-threose, m-cresol and oxoproline, with creatine exhibiting maximum integrated classification performance (VIP = 1.537).

**Fig. 5:**
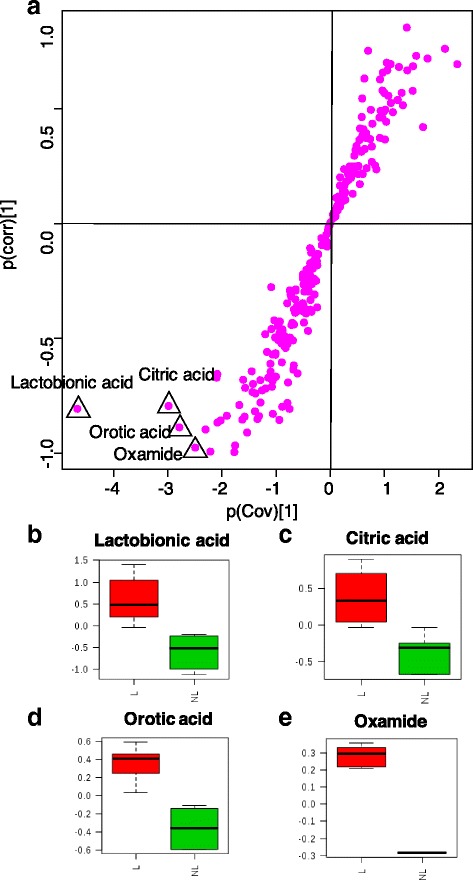
Potentially differentially expressed metabolites extracted by the S-plot between the lactation and non-lactation groups. **a** S-Plot, 4 metabolites are highlighted. These are shown in **b-e**. **b** Log normalized relative concentration of the 2 groups for lactobionic acid; **c** Log normalized relative concentration of the 2 groups for citric acid; **d** Log normalized relative concentration of the 2 groups for orotic acid; **e** Log normalized relative concentration of the 2 groups for oxamide; L: lactation group, NL: non-lactation group. The x-axis, p(cov), in figure a is a visualization of the contribution (covariance) to the module variables, and the y-axis, p(corr), in figure a is a visualization of the reliability (correlation) of the module. The preferred selection of the potential biomarkers is high covariance combined with high correlation. The red bar represents the lactation group, and the green bar represents the non-lactation group

In total, 73 differentially expressed metabolites were identified to in MG tissues between the lactating and non-lactating cows (VIP > 1, *P* < 0.05 & Q < 0.10), with 54 significantly higher and 19 significantly lower relative concentration metabolites in the lactating cows (Additional file 1: Table S4). The S-plot showed the key metabolites (Fig. 5). On the left-hand side of the S-plot, 4 variables with strong model contribution and high statistical reliability were explored as potential biomarkers to characterize the metabolic discriminations between lactation and non-lactation: lactobionic acid (P_cov_ = −4.669, P_corr_ = −0.806), citric acid (P_cov_ = −2.983, P_corr_ = −0.793), orotic acid (P_cov_ = −2.786, P_corr_ = −0.887) and oxamide (P_cov_ = −2.491, P_corr_ = −0.975).

### KEGG pathway analysis

**Fig. 6:**
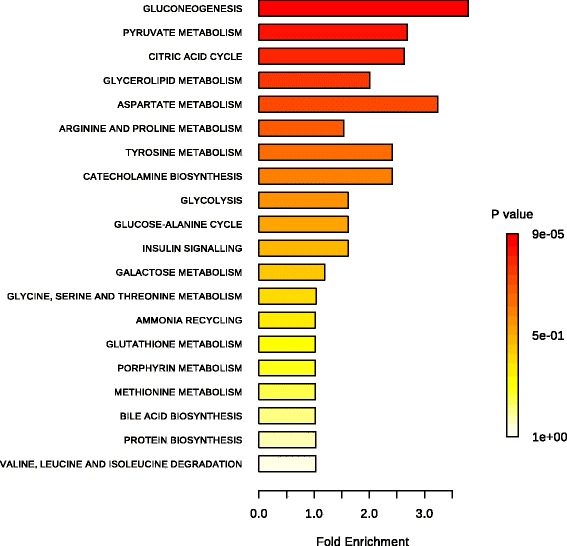
Functional enrichment of the different pathways identified by shared metabolites in the rumen fluid, serum, milk and urine. The bar chart size indicates the pathway enrichment, and the color density represents the difference. Pathways with a larger size and darker red color are the more enriched pathways

**Fig. 7:**
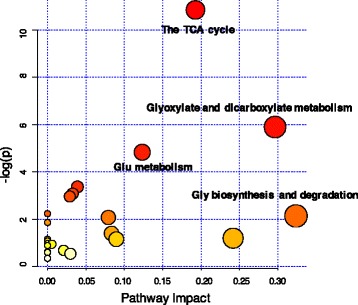
The metabolome view map of the functional impact pathways identified in the mammary gland from lactating and non-lactating cows. The x-axis represents the pathway enrichment and the y-axis represents the impact pathway. Larger sizes and darker colors represent the higher pathway enrichment and higher impact pathway values, respectively

**Fig. 8:**
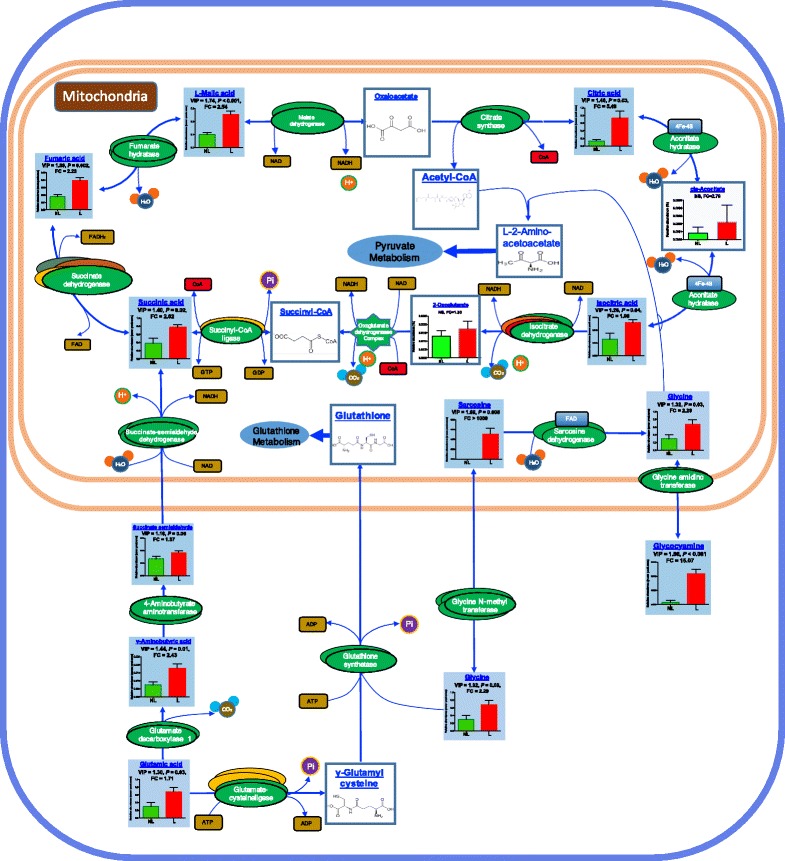
Integrated metabolomics of the lactating vs non-lactating mammary gland tissue. The figure combines the TCA cycle, glutamate metabolism, glycine biosynthesis and degradation pathways together with the detailed reactions, compounds, enzymes and energy carriers. The differentially expressed metabolites are illustrated by a column chart of 6 lactating cows and 6 non-lactating cows with the VIP (variable importance projection) and *P* values and fold changes between the groups

Overall, 20 pathways were obtained when the 33 shared metabolites from the four biofluids were imported into the KEGG analysis. Figure 6 shows the functional enrichment of the different pathways. The most enriched functional pathways included gluconeogenesis (FDR < 0.001, FE = 3.79), pyruvate metabolism (FDR = 0.003, FE = 2.68), the citric acid cycle (FDR = 0.007, FE = 2.63), glycerolipid metabolism (FDR = 0.017, FE = 2.01), and aspartate metabolism (FDR = 0.021, FE = 3.24). For the MG metabolomics, significantly higher abundance metabolites were involved in the 22 pathways in the lactation group (Additional file 1: Table S6). The key functional impact of the pathways is illustrated in Fig. 7. The results of the enrichment and impact pathways demonstrated that there were four significantly up-regulated pathways in the lactating cows: the tricarboxylic acid cycle (TCA), glyoxylate and dicarboxylate metabolism, glutamate metabolism, and glycine biosynthesis and degradation (*P* < 0.05, impact value >0.10). The integrated overview pathway map combined the above four key cellular pathways with the corresponding most significant and relevant metabolites (Fig. 8). Among these were seven metabolites that are involved in the TCA cycle, glyoxylate and dicarboxylate pathway, three metabolites that are involved in the glutamate metabolism pathway and three metabolites that are involved in the glycine biosynthesis and degradation pathway.

## Discussion

Remarkable progress has been made in our understanding of dairy cow metabolism, especially of the nutrient requirements of the biochemical reactions and functions [22]. However, the complex metabolic mechanisms that occur during lactation are still considered a “black box” due to our lack of in-depth understanding [23]. The increasing application of the -omics technologies (i.e., metagenomics, transcriptomics, proteomics and metabolomics) have become a powerful tool leading to the understanding of this “black box” [24]. Indeed, these -omics technologies are being used to evaluate the physiological and molecular changes of dairy cows under different environments, such as varied diets and management. The inherent metabolic status during lactation and the difference in the non-lactation period play a vital role in the lactation physiology, which determines the milk production, udder health and dairy sustainability [2]. Very little information is available on the metabolomics profiles of these two stages in the dairy cows. To our knowledge, this paper is the first to address the lactation-related metabolic profiles by using tissue and biofluid metabolomics. Both the overall lactation-related metabolism (variation and coordinated metabolic mechanism based on four biofluids’ metabolomics) and partial metabolism (comparison metabolomics in MGs between lactating and non-lactating cows) provide novel insights and a greater understanding of the metabolic mechanism of lactation.

For dairy cows, common biofluids include the rumen fluid, blood, milk and urine. The rumen fluid metabolites are critical for microbial metabolism and growth, host nutrient utilization and health, all of which can influence milk production [25]. Blood is an intermediary biofluid that consists of metabolites that are excreted from the body and links different organs and tissues [26]. The milk metabolomics reflect the product quality, metabolic processes and status of MG [27]. Urine is rich in metabolites and contains many biochemical pathway signals [28]. It is easy to distinguish the rumen fluid, serum, milk and urine based on their physical characteristics, such as the color and odor. However, the metabolic relationships, which may contain subtle metabolic profiling for the whole body, are not well understood. According to the metabolic flow in the dairy cow, the nutrients in the diet are first ingested and digested in the rumen, absorbed or passed through the rumen, transferred into blood in the small intestines, and then allocated to and utilized in the different organs, such as the MG. Waste materials are then extracted from the body through the urine [24]. It is reasonable to consider that the metabolites in the rumen fluid and serum are similar, whereas those in the urine are the most different from the other biofluids. In this study, creatine was characterized as an important metabolite that crosses four biofluids that have potential associations with lactation performance and health in dairy cows. Creatine is an important intermediate metabolite in the energy reactions, and its phosphorylated form is an important metabolite that is part of the energy shuttle [29]. In the reverse reaction, a high-energy phosphate group, such as ATP, is transferred to creatine to form phosphocreatine and ADP. There are two ways in which creatine may accumulate in animals due to the continual replacement of lost creatinine [30]: diet and de novo synthesis. Creatine is mainly synthesized in the liver from three amino acids: arginine, glycine and methionine. It is generally used by tissues, absorbed into blood and excreted in urine [31]. The relative concentration across the four biofluids from high to low were serum > urine > milk > rumen fluid, which represented the basic metabolic distribution of this metabolite. Milk creatine can help infants who suffer from defects in the arginine:glycine amidinotransferase and glycine amidinotransferase [32]. It is reported that more creatine is needed to ease the serious negative energy balance in dairy cows [33]. The creatine content is decreased in cows with ovarian inactivity, which may be related to the increased energy consumption in inactive cows [34]. Additionally, creatine was identified as a potential biomarker in diagnosing the heat stress status in dairy cows, which may be attributed to the phosphocreatine in the muscle tissue that have been mobilized for energy supply [35].

MSEA is a group-based approach that does not require the pre-selection of metabolites by using an arbitrary threshold. The key idea behind MSEA is to directly investigate the enrichment of pre-defined groups of functionally related metabolites (or metabolites sets) instead of individual metabolites, which have proven to be successful in deriving new information from the untargeted metabolomics studies [18]. In this study, all of the shared metabolites in the four biofluids were evaluated together, and their related biological information was incorporated into the results. For the mid-lactation dairy cows, the most active and important pathways, including gluconeogenesis, pyruvate metabolism, the TCA cycle, glycerolipid metabolism and aspartate metabolism, which were identified, reflected and confirmed by the overall metabolomics profiles of the four biofluids. Among them, gluconeogenesis, pyruvate metabolism and the TCA cycle are the key pathways of energy metabolism. Gluconeogenesis generates glucose from lactate, glycerol and glucogenic amino acids [36]. Glucose is extremely important for milk synthesis. In dairy cows, gluconeogenesis mostly occurs in the liver and provides up to 90% of the glucose required for host maintenance and production [37]. Pyruvate is the starting point of gluconeogenesis and the end product for glycolysis, and it can also be generated by the transamination of alanine [38]. Almost all of the dietary carbohydrates are fermented to volatile fatty acids (acetate, propionate and butyrate) in the rumen of the dairy cows, with propionate as the predominant substrate for gluconeogenesis [39]. Pyruvate is an important intermediate metabolite for the generation of propionate from the succinic pathway or the lactate pathway [40]. It can be converted by the pyruvate dehydrogenase complex into acetyl-CoA, which can then enter the TCA cycle. The TCA cycle plays a central role in cellular respiration and the supply of energy to all living cells [41], which is of paramount significance to the cell’s metabolic efficiency and, therefore, to the cow’s metabolism and production [42]. Insufficient energy is highly associated with ketosis (a severe metabolic disease) and can lead to decreased milk production [43]. In the glycerolipid metabolism pathway, glycerol can be converted into glucose by the liver and provide energy for cellular metabolism and lactation maintenance [44]. Aspartate is a precursor of many compounds that are involved in cellular signaling, such as N-acetyl-aspartate. It is also a metabolite in the urea cycle and participates in gluconeogenesis in dairy cows [45]. Based on the overview map of the KEGG pathway, these 5 key metabolic pathways were closely integrated together, suggesting that the carbohydrate pathways and energy pathways play vital roles in regulating lactation maintenance for the dairy cow.

Compared to the increase in the human breast metabolomics studies, to our knowledge, only 1 study has reported the goat MG secretory tissue metabolomics using NMR and identified 46 metabolites, with lactose, glutamate, glycine and lactate being the most abundant [46]. In our study, the most abundant metabolites in the lactating dairy cow MGs were lactic acid, lactobionic acid, oxoproline, alanine, diglycerol, glycine, citric acid, creatine and glutamic acid, of which lactobionic acid and oxoproline were first identified following other studies [27, 47]. The milk metabolomics of the dairy cows mainly focused on the composition and phenotype-related analyses, including distinguishing the formula milk from breast milk [48], comparing the colostrum and milk [49], discerning Holstein cows and other minor dairy animals [27], and analyzing the relationship with heat stress [9] or methane emission [42]. In this study, we also compared the similarities and differences between MG and milk metabolomics from the same lactating dairy cows. However, the four potential biomarkers (citric acid, lactobionic acid, oxamide, and orotic acid), including those metabolites that were found specifically in the MG from lactating cows, were also identified in the milk, which indicates that milk better reflects the physiological status of the MG compared to the non-lactating cows.

In this study, the S-plot combined with the impact pathway analysis was proven to be an effective and easy way to screen most statistically significant metabolites and functional impact pathways. The S-plot indicated that lactobionic acid, citric acid, orotic acid and oxamide (identified only in the lactation group) play vital roles in discriminating between lactating and non-lactating cows. The TCA cycle, glutamate metabolism and glycine metabolism pathways are the most important and work together in the MG for lactation initiation. Milk component synthesis and secretion is the main transition in lactation initiation. Lactobionic acid is the intermediate metabolite in the lactose biosynthesis pathway, is produced by lactose oxidation and has strong mineral complex properties that serve as promising bioactive ingredients in human nutrition [50]. Citric acid is formed in the TCA cycle or from the diet and participates in the intermediate metabolism of carbohydrate oxidation in animal tissues [51]. The level of citric acid is significantly higher in the lactating dairy cows and may enhance energy by participating in the TCA cycle. Orotic acid is found at a high concentration in bovine milk and almost exclusively originates in the MG cells [52]. Orotic acid has been applied to improve athletic performance and body composition, and it has proven to enhance ATP [53]. Oxamide is a diamide that is derived from oxalic acid and participates in the TCA cycle. In this study, oxamide and other secondary metabolites were significantly higher in the lactating dairy cows, indicating that the TCA cycle is more active during lactation initiation. Oxamide was also identified in the milk in the lactation group, suggesting that the TCA cycle is up-regulated in the lactation group.

Moreover, the functional impact pathway analysis showed that the TCA cycle pathway had the highest *P* value between the groups, which was mainly attributed to the 7 up-regulated metabolites of the 10 main substrates in the TCA cycle. It has been reported that a critical energy output is required during the initiation of lactation to support milk synthesis and secretion of mammary epithelial cells [54]. Figure 8 depicts the integration of the most significant pathways and the most significant and relevant metabolites, which suggests the different metabolic mechanisms in the MG of the lactating cows compared to the non-lactating cows. Therefore, the downstream metabolites in the glutamic acid pathway entered the TCA cycle to generate succinate in the mitochondria through succinate semialdehyde, thereby releasing NADH. It is well known that NADH is used by the oxidative phosphorylation pathway to generate ATP [55], which can transport chemical energy within the cells for metabolism and provide a larger amount of energy for lactation maintenance and related biological processes. In addition to supplying energy sources, the TCA cycle integrates many other pathways to unify carbohydrate, protein and fat metabolism [56]. For example, citric acid can be transported out of the mitochondrion to produce cytosolic acetyl-CoA for fatty acid and cholesterol (which further synthesizes steroid hormones and vitamin D) synthesis, which is also very important for lactation maintenance [57]. Another intermediate of glutamate metabolism, γ-glutamyl-cysteine, is used to synthesize glutathione, which is vital for preventing damage to important cellular components of the mammary epithelial cells [58]. As a prime metabolic source of glutathione, creatine, purines and serine and a protein constituent in the lactating mammary epithelial cells, glycine plays a significant role in various biological processes [59]. With respect to the Gly pathway, two metabolites were highlighted: sarcosine and glycocyamine. Sarcosine is an intermediate and byproduct in glycine synthesis and degradation [60]. It can be rapidly degraded into glycine in the mitochondria, which serves as a substrate along with acetyl-CoA to generate L-2-aminoacetoacetate and interacts with the pyruvate metabolism pathway. Glycocyamine is a precursor of creatine, which serves as an essential substrate for muscle energy metabolism and has been used in the feed additive industry to improve production quality [61]. The above results suggest that major energy-related metabolic changes occur in the MG of lactating cows at the initiation of lactation to accommodate the demand for increased milk synthesis nutrients.

## Conclusions

Using MG tissue and multi-biofluid metabolomics, the metabolic mechanisms of inherent lactation metabolic patterns and differences with non-lactation periods were addressed in this study. Based on the HCA of the 33 shared metabolites, the rumen fluid and serum were highly correlated and grouped together with milk. Creatine was characterized as the key metabolite to explain the biological variation among the four biofluids. For the lactating cows, lactobionic acid, citric acid, orotic acid and oxamide were identified as potential biomarkers among 54 differentially expressed metabolites. Gluconeogenesis, pyruvate metabolism, the TCA cycle, glycerolipid metabolism and the aspartate metabolism pathways were the most functionally enriched pathways. Integrated analysis of the differentially expressed metabolites involved in the TCA cycle, glutamate metabolism and glycine biosynthesis and degradation pathways revealed the probable key metabolic mechanism in the MG during lactation. Overall, our results provide a better physiological understanding of the lactation metabolism of mid-lactation dairy cows, which can help elucidate the regulated metabolic strategies for the lactating dairy cows in the future. More importantly, the combined application of the multi-biofluids and the tissue metabolomics in this study provide new insights into addressing complex biological questions. Further studies are required to validate the potential biomarkers and pathways identified in this study.

## Additional file

Additional file 1: Table S1. Ingredients and nutrient composition of the experimental diet. **Table S2.** The relative abundance of mutual metabolites in rumen fluid, serum, milk and urine. **Table S3.** Mutual metabolites identified in the mammary gland and milk in lactation group. **Table S4** The metabolites identified in the lactation (N) and non-lactation (NL) groups. **Table S5.** Differentially expressed metabolites among the 4 biofluids identified by one-way ANOVA and post-hoc analysis. **Table S6.** Significantly up-regulated pathways in lactation group. (DOCX 70 kb)

## Abbreviations

FDR: False discovery rate
FE: Fold enrichment
GC-TOF/MS: Gas chromatography-time of flight/mass spectrometry
HCA: Hierarchical cluster analysis
MG: Mammary gland
MSEA: metabolite set enrichment analysis
OPLS: Orthogonal projections to latent structures
PCA: Principal component analysis
PLS-DA: Partial least squares discriminant analysis
TCA: Tricarboxylic acid cycle
VIP: Variable importance projection
