# Supplementary material for: Lactation-related metabolic mechanism investigated based on mammary gland metabolomics and 4 biofluids’ metabolomics relationships in dairy cows
Source: BMC Genomics. 2017 Dec 2;18:936. doi: 10.1186/s12864-017-4314-1 (PMC5712200; doi:10.1186/s12864-017-4314-1)
Supplement: Additional file 1: Table S1. — Ingredients and nutrient composition of the experimental diet. Table S2. The relative abundance of mutual metabolites in rumen fluid, serum, milk and urine. Table S3. Mutual metabolites identified in the mammary gland and milk in lactation group. Table S4 The metabolites identified in the lactation (N) and non-lactation (NL) groups. Table S5. Differentially expressed metabolites among the 4 biofluids identified by one-way ANOVA and post-hoc analysis. Table S6. Significantly up-regulated pathways in lactation group. (DOCX 70 kb) [file 12864_2017_4314_MOESM1_ESM.docx]

**Lactation-related metabolic mechanism investigated based on mammary gland metabolomics and 4 biofluids metabolomics relationships in dairy cows**

Hui-Zeng Sun, Kai Shi, Xue-Hui Wu, Ming-Yuan Xue, Zi-Hai Wei, Jian-Xin Liu, Hong-Yun Liu*

Institute of Dairy Science, MoE Key Laboratory of Molecular Animal Nutrition, College of Animal Sciences, Zhejiang University, Hangzhou 310058, P.R. China

*Corresponding author: hyliu@zju.edu.cn

**Table S1** Ingredients and nutrient composition of the experimental diet

| Item | % as a DM basis |
| --- | --- |
| *Ingredients* | |
| Ground corn grain | 27 |
| Wheat bran | 8.1 |
| Soybean meal | 7.5 |
| Cottonseed meal | 4.3 |
| Beet pulp | 4.1 |
| Corn silage | 15 |
| Alfalfa hat | 23 |
| Chinese wild ryegrass hay | 7 |
| Urea | 1 |
| Premix | 4 |
| *Nutrients* | |
| DM | 52.9 |
| OM | 92.0 |
| CP | 16.7 |
| NDF | 31.1 |
| ADF | 18.9 |
| NFC | 40.6 |
| Pectin | 14.8 |
| Ca | 0.91 |
| P | 0.47 |
| NE_L_ , Mcal/kg | 1.57 |

**Table S2** The relative abundance of mutual metabolites in rumen fluid, serum, milk and urine

| **Metabolites** | **Relative abundance^1^** | | | |
| --- | --- | --- | --- | --- |
|  | **Rumen fluid** | **Serum** | **Milk** | **Urine** |
| 2,4-diaminobutyric acid | 0.01619 | 0.01483 | 0.00437 | 0.00811 |
| 2-hydroxybutanoic acid | 0.00027 | 0.00116 | 0.06607 | 0.01229 |
| 4-androsten-11beta-ol-3,17-dione | 0.00062 | 0.00006 | 0.02161 | 0.00281 |
| 5-aminovaleric acid | 0.00656 | 0.00202 | 0.00030 | 0.01598 |
| 5-methoxytryptamine | 0.00051 | 0.00017 | 0.00120 | 0.00288 |
| alanine | 0.43018 | 0.03861 | 0.00052 | 1.54952 |
| aminomalonic acid | 0.00088 | 0.00273 | 0.02326 | 0.00289 |
| asparagine | 0.00058 | 0.00082 | 0.14278 | 0.00197 |
| conduritol b epoxide | 0.00064 | 0.01164 | 0.17820 | 0.09011 |
| creatine degr | 0.00066 | 0.00297 | 0.31985 | 0.00537 |
| fumaric acid | 0.00161 | 0.00098 | 0.00511 | 0.00064 |
| glucose | 0.08001 | 0.11141 | 0.16257 | 0.00634 |
| glycerol | 0.15453 | 0.00076 | 2.29554 | 0.01687 |
| glycine | 0.01516 | 0.03925 | 1.40907 | 0.01592 |
| hydroxylamine | 3.52400 | 0.00071 | 0.52257 | 4.11587 |
| Isoleucine | 0.00060 | 0.00021 | 0.14136 | 0.04024 |
| lactic acid | 0.03911 | 0.24309 | 6.68497 | 0.02637 |
| lactose | 0.01059 | 0.03823 | 53.4649 | 2.91377 |
| L-malic acid | 0.01239 | 0.00029 | 24.0257 | 0.00703 |
| L-threose | 0.00107 | 0.00112 | 0.24884 | 0.03744 |
| lyxose | 0.27525 | 0.00087 | 2.83806 | 0.00003 |
| malonic acid | 0.00578 | 0.00080 | 0.05603 | 0.02746 |
| m-cresol | 0.00262 | 0.00212 | 0.01371 | 2.14614 |
| methylmalonic acid | 0.00299 | 0.00021 | 0.01218 | 0.04872 |
| N-methyl-L-glutamic acid | 0.00074 | 0.00036 | 0.00116 | 0.07523 |
| noradrenaline | 0.00232 | 0.00030 | 0.01277 | 0.00955 |
| norleucine | 0.00025 | 0.00015 | 4.81744 | 0.01714 |
| oxalic acid | 0.00080 | 0.00029 | 4.39164 | 0.01675 |
| oxoproline | 0.00119 | 0.00006 | 0.04568 | 0.01019 |
| phenylethylamine | 0.00095 | 0.00151 | 220.375 | 0.00360 |
| phosphate | 0.43844 | 0.07916 | 0.01772 | 13.3553 |
| prostaglandin E2 | 0.00505 | 0.00111 | 0.05620 | 0.00225 |
| thymol | 0.00073 | 0.00012 | 0.00014 | 0.00528 |

^1^Relative abundance = Mean peak area

**Table S3** Mutual metabolites identified in the mammary gland and milk in lactation group

| lactic acid | D-galacturonic acid | phosphate |
| --- | --- | --- |
| Lactobionic Acid | N-Methyl-DL-alanine | Lyxose |
| oxoproline | hydroxylamine | Maleimide |
| alanine | Myristic Acid | 5-Aminovaleric acid |
| Diglycerol | fructose | citrulline |
| myo-inositol | xylose | maleic acid |
| citric acid | Citraconic acid | Mono(2-ethylhexyl)phthalate |
| glycine | Aminomalonic acid | alpha-ketoglutaric acid |
| creatine | D-erythronolactone | 1-Methylhydantoin |
| 2-Deoxyerythritol | conduritol b epoxide | Cortexolone |
| proline | 4-hydroxybutyrate | shikimic acid |
| glutamic acid | fucose | L-Threose |
| palmitic acid | creatine degr | Atropine |
| L-Malic acid | uracil | Zymosterol |
| valine | lauric acid | Methylmalonic acid |
| Ethanolamine | sorbitol | isocitric acid |
| beta-Glycerophosphoric acid | asparagine | glycocyamine |
| Glucose-1-phosphate | Hippuric acid | Prostaglandin E2 |
| Isoleucine | cis-gondoic acid | oxalic acid |
| putrescine | 2-hydroxybutanoic acid | 2-Amino-1-phenylethanol |
| putrescine | glucose-6-phosphate | Gluconic lactone |
| Orotic acid | succinate semialdehyde | threonine |
| malonic acid | pantothenic acid | 5-Methoxytryptamine |
| beta-Mannosylglycerate | 3-Hydroxypyridine | D-(glycerol 1-phosphate) |
| Methyl Phosphate | 2,2-Dimethylsuccinic Acid | melibiose |
| glutamine | heptadecanoic acid | 6-Hydroxynicotinic acid |
| 3-hydroxybutyric acid | ribulose-5-phosphate | D-erythro-sphingosine |
| O-Phosphorylethanolamine | oxamide | 3-Hydroxynorvaline |
| phosphomycin | 3-Methylamino-1,2-propanediol | Threitol |
| fumaric acid | 2-ketobutyric acid | Norleucine |
| lactose | cycloleucine | Erythrose |
| lysine | linoleic acid | 3,4-dihydroxybenzoic acid |
| phenylalanine | 2-Monopalmitin | cis-Phytol |
| Dehydroascorbic Acid | Carnitine | Galactinol |
| 3-Hydroxypropionic acid | 2,3-Dihydroxypyridine | Glucoheptonic acid |
| tryptophan | aspartic acid | glucose |
| 1,5-Anhydroglucitol | D-Glyceric acid | Thymol |
| N-Acetyl-beta-D-mannosamine | benzoic acid | serine |
| L-glutamic acid | Pyruvic acid | succinic acid |
| tyrosine |  |  |

**Table S4 The metabolites identified in the lactation (N) and non-lactation (NL) groups**

| **Peak** | **Similarity** | **R.T.** | **Mass** | **VIP** | **P** | **Q** | **Fold Change** |
| --- | --- | --- | --- | --- | --- | --- | --- |
| zymosterol | 712 | 28.360 | 129 | 1.057 | 0.096 | 0.127 | 2.490 |
| xylose | 920 | 15.162 | 103 | 1.469 | 0.012 | 0.045 | 1.480 |
| xanthine | 873 | 18.892 | 353 | 1.334 | 0.027 | 0.071 | 2.164 |
| valine | 968 | 9.499 | 144 | 1.538 | 0.007 | 0.035 | 0.537 |
| uridine | 806 | 22.618 | 224 | 0.685 | 0.302 | 0.230 | 1.420 |
| uric acid | 809 | 19.639 | 441 | 1.439 | 0.014 | 0.050 | 1.661 |
| uracil | 936 | 11.176 | 99 | 0.209 | 0.759 | 0.398 | 1.144 |
| tyrosine | 942 | 18.127 | 218 | 1.515 | 0.008 | 0.037 | 0.452 |
| tryptophan | 818 | 20.708 | 202 | 1.335 | 0.027 | 0.071 | 0.612 |
| trehalose | 216 | 24.789 | 93 | 0.890 | 0.200 | 0.182 | >1000 |
| Thymol | 314 | 10.935 | 257 | 1.175 | 0.087 | 0.122 | <0.001 |
| thymine | 324 | 11.916 | 113 | 0.531 | 0.429 | 0.281 | 1.418 |
| thymidine 5'-monophosphate | 211 | 25.818 | 243 | 0.268 | 0.694 | 0.377 | 0.680 |
| threonine | 540 | 10.595 | 146 | 1.115 | 0.105 | 0.131 | >1000 |
| threonic acid | 869 | 13.794 | 292 | 1.229 | 0.046 | 0.092 | 0.597 |
| threo-beta-hyrdoxyaspartate | 65 | 14.267 | 142 | 0.634 | 0.341 | 0.244 | 1.330 |
| threitol | 692 | 13.248 | 217 | 1.509 | 0.009 | 0.037 | 0.352 |
| terephthalic acid | 666 | 16.655 | 295 | 1.427 | 0.033 | 0.078 | <0.001 |
| taurine | 309 | 15.392 | 137 | 0.243 | 0.721 | 0.386 | 0.868 |
| tartronic acid | 504 | 11.681 | 147 | 1.645 | 0.003 | 0.026 | 1.680 |
| sulfuric acid | 625 | 8.813 | 147 | 0.654 | 0.326 | 0.239 | 0.836 |
| sucrose-6-Phosphate | 357 | 26.663 | 171 | 1.654 | 0.010 | 0.040 | 26.325 |
| succinic acid | 948 | 10.865 | 147 | 1.395 | 0.019 | 0.058 | 2.017 |
| succinate semialdehyde | 362 | 9.123 | 132 | 1.179 | 0.058 | 0.104 | 1.367 |
| stigmasterol | 544 | 28.888 | 109 | 1.372 | 0.042 | 0.088 | >1000 |
| stearic acid | 910 | 20.964 | 117 | 0.177 | 0.799 | 0.411 | 0.889 |
| spermidine | 505 | 21.052 | 144 | 0.404 | 0.550 | 0.326 | 0.706 |
| sorbose | 953 | 17.416 | 103 | 0.766 | 0.270 | 0.217 | 0.600 |
| sorbitol | 837 | 18.089 | 103 | 0.159 | 0.816 | 0.416 | 1.108 |
| sophorose | 860 | 25.202 | 361 | 1.690 | 0.007 | 0.036 | 11.735 |
| shikimic acid | 641 | 16.740 | 316 | 0.041 | 0.954 | 0.454 | 0.980 |
| serine | 919 | 11.468 | 204 | 0.988 | 0.124 | 0.139 | 0.550 |
| sarcosine | 467 | 8.246 | 101 | 1.686 | 0.008 | 0.037 | >1000 |
| salicin | 628 | 22.911 | 217 | 1.593 | 0.004 | 0.031 | 4.508 |
| ribulose-5-phosphate | 693 | 19.784 | 357 | 0.153 | 0.823 | 0.418 | 1.117 |
| ribose-5-phosphate | 654 | 19.686 | 315 | 1.261 | 0.040 | 0.085 | 2.058 |
| ribose | 926 | 15.336 | 103 | 0.258 | 0.705 | 0.381 | 0.935 |
| ribonic acid, gamma-lactone | 377 | 15.320 | 328 | 1.347 | 0.025 | 0.068 | 0.494 |
| raffinose | 622 | 29.219 | 204 | 0.122 | 0.858 | 0.428 | 1.133 |
| pyruvic acid | 788 | 7.131 | 174 | 0.281 | 0.679 | 0.372 | 0.904 |
| pyrrole-2-Carboxylic Acid | 540 | 11.353 | 240 | 0.678 | 0.307 | 0.232 | 0.371 |
| pyrophosphate | 847 | 15.135 | 451 | 0.666 | 0.317 | 0.236 | 0.777 |
| pyrogallol | 230 | 13.758 | 114 | 0.939 | 0.176 | 0.170 | <0.001 |
| pyridoxal phosphate | 314 | 21.819 | 211 | 0.650 | 0.347 | 0.246 | 1.348 |
| putrescine | 952 | 16.053 | 174 | 0.083 | 0.903 | 0.441 | 0.962 |
| purine riboside | 501 | 22.132 | 246 | 1.169 | 0.088 | 0.123 | <0.001 |
| prostaglandin E2 | 294 | 25.350 | 330 | 0.471 | 0.484 | 0.302 | 1.323 |
| proline | 980 | 10.669 | 142 | 1.223 | 0.048 | 0.094 | 0.659 |
| picolinic acid | 298 | 11.188 | 180 | 0.103 | 0.879 | 0.434 | 1.106 |
| piceatannol | 303 | 23.313 | 301 | 0.635 | 0.363 | 0.252 | >1000 |
| phosphomycin | 367 | 11.463 | 340 | 0.634 | 0.341 | 0.244 | 1.446 |
| phosphate | 695 | 10.311 | 373 | 0.667 | 0.333 | 0.242 | 3.599 |
| phloroglucinol | 323 | 14.956 | 326 | 0.991 | 0.122 | 0.139 | 0.472 |
| phenylphosphoric acid | 500 | 14.487 | 211 | 1.347 | 0.025 | 0.068 | 1.601 |
| phenylalanine | 956 | 14.804 | 218 | 1.017 | 0.112 | 0.134 | 0.529 |
| phenyl beta-D-glucopyranoside | 501 | 21.393 | 259 | 1.472 | 0.027 | 0.071 | <0.001 |
| pelargonic acid | 609 | 11.511 | 215 | 0.466 | 0.489 | 0.304 | 2.248 |
| pantothenic acid | 873 | 18.630 | 103 | 0.419 | 0.535 | 0.321 | 1.121 |
| palmitic acid | 959 | 19.181 | 117 | 0.418 | 0.537 | 0.321 | 0.827 |
| palatinitol | 661 | 26.072 | 202 | 2.026 | 0.000 | 0.008 | >1000 |
| oxoproline | 887 | 13.598 | 156 | 0.441 | 0.523 | 0.317 | 1.122 |
| oxamide | 561 | 10.937 | 102 | 1.961 | 0.000 | 0.014 | >1000 |
| oxalic acid | 406 | 8.280 | 168 | 0.160 | 0.814 | 0.415 | 0.943 |
| orotic acid | 917 | 16.095 | 254 | 1.727 | 0.006 | 0.034 | 23.96 |
| ornithine | 758 | 14.647 | 142 | 1.511 | 0.009 | 0.037 | 0.346 |
| O-phosphoserine | 418 | 17.051 | 356 | 0.927 | 0.152 | 0.156 | 0.471 |
| O-phosphorylethanolamine | 941 | 16.524 | 174 | 0.314 | 0.644 | 0.360 | 0.776 |
| O-phosphonothreonine | 406 | 16.302 | 266 | 0.948 | 0.142 | 0.150 | 0.434 |
| oleic acid | 967 | 20.740 | 117 | 1.140 | 0.089 | 0.123 | 0.569 |
| octanal | 107 | 8.139 | 273 | 0.799 | 0.224 | 0.194 | 1.557 |
| octadecanol | 828 | 20.186 | 327 | 1.838 | 0.002 | 0.025 | <0.001 |
| norvaline | 408 | 9.919 | 100 | 0.972 | 0.131 | 0.143 | 2.629 |
| norleucine | 246 | 10.955 | 260 | 0.443 | 0.512 | 0.313 | 1.941 |
| N-oleoyldopamine | 548 | 25.457 | 95 | 0.883 | 0.204 | 0.184 | >1000 |
| N-methyl-DL-alanine | 955 | 8.880 | 130 | 0.410 | 0.544 | 0.324 | 0.803 |
| nicotinamide | 915 | 13.215 | 179 | 0.896 | 0.168 | 0.166 | 1.668 |
| nicotianamine | 337 | 23.744 | 246 | 1.151 | 0.094 | 0.126 | <0.001 |
| N-ethylmaleamic acid | 109 | 12.905 | 281 | 1.039 | 0.133 | 0.144 | >1000 |
| neohesperidin | 372 | 22.865 | 204 | 1.262 | 0.054 | 0.100 | 4.190 |
| N-cyclohexylformamide | 108 | 9.591 | 207 | 0.738 | 0.287 | 0.224 | 0.722 |
| N-acetyl-D-galactosamine | 946 | 19.462 | 87 | 1.381 | 0.038 | 0.083 | 5.348 |
| N-acetyl-beta-D-mannosamine | 729 | 19.649 | 202 | 1.074 | 0.113 | 0.135 | 0.370 |
| myristic Acid | 809 | 17.235 | 132 | 0.573 | 0.406 | 0.271 | 1.471 |
| myo-inositol | 911 | 19.610 | 191 | 0.991 | 0.122 | 0.139 | 0.790 |
| monostearin | 536 | 25.141 | 399 | 0.583 | 0.383 | 0.261 | 0.662 |
| mono(2-ethylhexyl)phthalate | 200 | 19.918 | 267 | 0.834 | 0.203 | 0.183 | 1.555 |
| methylmalonic acid | 698 | 9.412 | 147 | 0.328 | 0.629 | 0.354 | 1.743 |
| methyl-beta-D-galactopyranoside | 645 | 17.285 | 204 | 1.363 | 0.043 | 0.088 | 5.241 |
| methyl Phosphate | 881 | 8.947 | 241 | 0.048 | 0.944 | 0.452 | 1.016 |
| methyl Palmitoleate | 510 | 17.841 | 110 | 0.850 | 0.193 | 0.179 | 0.476 |
| methyl jasmonate 4 | 246 | 15.790 | 207 | 0.635 | 0.363 | 0.252 | <0.001 |
| methoxamedrine | 370 | 16.443 | 267 | 0.382 | 0.572 | 0.333 | 1.954 |
| methionine | 733 | 13.523 | 176 | 1.113 | 0.077 | 0.117 | 0.412 |
| melibiose | 559 | 25.810 | 204 | 1.062 | 0.095 | 0.126 | 0.163 |
| melezitose | 450 | 29.781 | 204 | 0.805 | 0.220 | 0.193 | 0.519 |
| maltose | 939 | 24.842 | 361 | 0.857 | 0.218 | 0.191 | <0.001 |
| malonic acid | 651 | 9.222 | 147 | 0.825 | 0.208 | 0.186 | 0.767 |
| maleimide | 849 | 7.812 | 154 | 0.901 | 0.165 | 0.164 | 0.692 |
| maleic acid | 47 | 10.727 | 341 | 1.027 | 0.107 | 0.132 | 1.363 |
| maleamate | 408 | 14.055 | 244 | 0.121 | 0.859 | 0.428 | 1.090 |
| lyxose | 709 | 15.023 | 103 | 1.437 | 0.032 | 0.077 | >1000 |
| lysine | 845 | 17.962 | 174 | 0.741 | 0.262 | 0.213 | 0.458 |
| L-threose | 688 | 12.605 | 147 | 1.710 | 0.007 | 0.035 | >1000 |
| Loganin | 649 | 26.141 | 202 | 1.449 | 0.028 | 0.073 | 0.046 |
| L-malic acid | 971 | 13.110 | 147 | 1.744 | 0.001 | 0.016 | 2.540 |
| linoleic acid methyl ester | 612 | 19.677 | 122 | 0.068 | 0.921 | 0.445 | 0.912 |
| linoleic acid | 809 | 20.671 | 337 | 0.220 | 0.746 | 0.394 | 0.919 |
| lignoceric acid | 632 | 25.542 | 132 | 1.000 | 0.119 | 0.137 | 2.132 |
| L-homoserine | 263 | 15.262 | 204 | 0.014 | 0.983 | 0.462 | 1.013 |
| L-glutamic acid | 772 | 13.665 | 174 | 0.969 | 0.132 | 0.144 | 1.487 |
| levoglucosan | 354 | 15.695 | 204 | 0.392 | 0.563 | 0.330 | 1.396 |
| leucine | 598 | 10.292 | 158 | 1.607 | 0.004 | 0.030 | 0.490 |
| L-cysteine | 892 | 13.950 | 220 | 0.745 | 0.259 | 0.212 | 1.316 |
| lauric acid | 869 | 15.106 | 117 | 0.271 | 0.697 | 0.378 | 1.160 |
| L-allothreonine | 942 | 11.805 | 219 | 0.916 | 0.157 | 0.160 | 0.585 |
| lactobionic Acid | 775 | 24.992 | 191 | 1.404 | 0.036 | 0.081 | 10.992 |
| lactic acid | 780 | 7.303 | 117 | 0.564 | 0.413 | 0.274 | 0.797 |
| lactamide | 485 | 8.433 | 220 | 0.179 | 0.792 | 0.409 | 1.058 |
| isopropyl-beta-D-thiogalactopyranoside | 230 | 19.421 | 310 | 1.807 | 0.000 | 0.013 | 2.446 |
| isoleucine | 949 | 10.598 | 158 | 1.023 | 0.109 | 0.133 | 0.545 |
| isocitric acid | 380 | 16.964 | 491 | 1.255 | 0.041 | 0.086 | 1.994 |
| inosine | 937 | 23.596 | 230 | 0.683 | 0.304 | 0.231 | 1.764 |
| indole-3-acetamide | 153 | 21.241 | 221 | 0.434 | 0.520 | 0.316 | 1.449 |
| iminodiacetic acid | 942 | 13.532 | 232 | 0.151 | 0.828 | 0.419 | 0.948 |
| hypoxanthine | 879 | 16.811 | 265 | 0.544 | 0.417 | 0.276 | 1.502 |
| hydroxylamine | 931 | 8.082 | 146 | 0.763 | 0.247 | 0.206 | 1.336 |
| hydrocortisone | 477 | 26.480 | 119 | 1.601 | 0.010 | 0.041 | 1.578 |
| hydrocinnamic acid | 370 | 12.327 | 104 | 0.684 | 0.303 | 0.230 | 3.964 |
| Hippuric acid | 827 | 17.306 | 105 | 1.368 | 0.022 | 0.064 | 2.843 |
| hexadecane | 330 | 14.548 | 113 | 0.706 | 0.286 | 0.223 | 1.583 |
| heptadecanoic acid | 926 | 20.080 | 117 | 0.151 | 0.828 | 0.419 | 1.116 |
| guanosine | 674 | 25.063 | 324 | 0.421 | 0.533 | 0.320 | 1.451 |
| guanine | 719 | 19.905 | 352 | 0.662 | 0.319 | 0.237 | 1.592 |
| guanidinosuccinic acid 4 | 329 | 19.517 | 383 | 1.024 | 0.128 | 0.141 | 1.913 |
| gly-pro | 175 | 18.753 | 293 | 0.114 | 0.867 | 0.430 | 0.979 |
| glycolic acid | 855 | 7.504 | 147 | 0.442 | 0.525 | 0.317 | 0.874 |
| glycocyamine | 203 | 15.935 | 180 | 1.865 | 0.000 | 0.008 | 15.07 |
| glycine-d5 | 492 | 10.765 | 110 | 0.407 | 0.547 | 0.325 | 0.740 |
| glycine | 957 | 10.778 | 174 | 1.321 | 0.029 | 0.073 | 2.287 |
| glutaric Acid | 887 | 12.065 | 147 | 1.033 | 0.126 | 0.140 | 0.525 |
| glutamine | 946 | 16.448 | 156 | 0.967 | 0.155 | 0.158 | 0.565 |
| glutamic acid | 942 | 14.704 | 246 | 1.303 | 0.032 | 0.077 | 1.711 |
| glucose-6-phosphate | 901 | 21.540 | 387 | 0.182 | 0.793 | 0.409 | 1.096 |
| glucose-1-phosphate | 607 | 16.363 | 217 | 1.092 | 0.084 | 0.121 | 1.731 |
| glucose | 692 | 17.626 | 179 | 0.635 | 0.363 | 0.252 | <0.001 |
| glucosaminic acid | 288 | 19.115 | 186 | 0.696 | 0.294 | 0.226 | 0.436 |
| gluconic lactone | 567 | 17.750 | 264 | 1.067 | 0.093 | 0.125 | 5.629 |
| gluconic acid | 887 | 18.698 | 147 | 0.026 | 0.970 | 0.458 | 1.012 |
| glucoheptonic acid | 469 | 21.039 | 196 | 1.188 | 0.083 | 0.120 | <0.001 |
| galactonic acid | 257 | 18.653 | 264 | 0.635 | 0.363 | 0.252 | >1000 |
| galactinol | 369 | 26.537 | 204 | 1.188 | 0.083 | 0.120 | 0.000 |
| fumaric acid | 938 | 11.350 | 245 | 1.692 | 0.002 | 0.021 | 2.231 |
| fucose | 838 | 16.030 | 117 | 0.725 | 0.292 | 0.226 | 0.486 |
| fructose-6-phosphate | 806 | 21.433 | 315 | 0.135 | 0.843 | 0.424 | 1.077 |
| fructose 2,6-biphosphate degr prod | 641 | 20.666 | 227 | 1.323 | 0.029 | 0.073 | 1.491 |
| fructose | 847 | 17.511 | 103 | 0.913 | 0.187 | 0.176 | 0.516 |
| ethanolamine | 867 | 10.219 | 174 | 0.497 | 0.460 | 0.293 | 1.201 |
| erythrose 4-phosphate | 150 | 17.722 | 284 | 0.847 | 0.194 | 0.179 | 3.136 |
| erythrose | 414 | 12.374 | 201 | 0.034 | 0.960 | 0.456 | 1.036 |
| D-Talose | 938 | 17.704 | 160 | 1.450 | 0.013 | 0.048 | 0.422 |
| diglycerol | 490 | 16.323 | 101 | 0.625 | 0.349 | 0.247 | 1.139 |
| D-glyceric acid | 864 | 11.054 | 189 | 1.217 | 0.073 | 0.114 | 2.898 |
| d-glucoheptose | 604 | 19.862 | 143 | 1.742 | 0.004 | 0.031 | 4.384 |
| D-galacturonic acid | 789 | 18.058 | 160 | 1.908 | 0.001 | 0.015 | 20.12 |
| D-erythro-sphingosine | 590 | 22.731 | 204 | 0.627 | 0.347 | 0.247 | 0.352 |
| D-erythronolactone | 407 | 14.459 | 103 | 1.842 | 0.002 | 0.024 | >1000 |
| dehydroascorbic Acid | 943 | 17.195 | 173 | 0.642 | 0.335 | 0.242 | 0.791 |
| dehydroabietic Acid | 286 | 22.510 | 294 | 0.610 | 0.361 | 0.251 | 1.201 |
| D-arabitol | 928 | 15.655 | 217 | 0.189 | 0.782 | 0.405 | 1.042 |
| D-(glycerol 1-phosphate) | 532 | 16.202 | 174 | 0.280 | 0.680 | 0.372 | 1.271 |
| cytosin | 473 | 13.613 | 240 | 0.268 | 0.693 | 0.377 | 1.578 |
| cytidine-monophosphate | 327 | 18.812 | 283 | 0.635 | 0.363 | 0.252 | <0.001 |
| cystine | 766 | 21.358 | 146 | 1.458 | 0.024 | 0.067 | 6.721 |
| cycloleucine | 648 | 11.557 | 156 | 1.323 | 0.029 | 0.073 | 1.587 |
| cyclic-GMP | 326 | 27.924 | 211 | 1.102 | 0.081 | 0.119 | 4.962 |
| creatine degr | 609 | 14.948 | 147 | 0.086 | 0.900 | 0.440 | 0.947 |
| creatine | 909 | 13.947 | 115 | 0.250 | 0.717 | 0.385 | 0.902 |
| corticosterone | 270 | 28.618 | 221 | 0.737 | 0.265 | 0.214 | 1.463 |
| cortexolone | 295 | 27.651 | 119 | 1.450 | 0.013 | 0.048 | 7.202 |
| conduritol b epoxide | 757 | 18.212 | 103 | 0.370 | 0.585 | 0.338 | 1.258 |
| citrulline | 713 | 16.954 | 157 | 1.482 | 0.011 | 0.042 | 0.327 |
| citric acid | 926 | 16.889 | 273 | 1.457 | 0.026 | 0.070 | 5.495 |
| citramalic acid | 196 | 12.899 | 203 | 0.277 | 0.691 | 0.376 | 0.790 |
| citraconic acid | 921 | 11.230 | 147 | 0.986 | 0.124 | 0.140 | 2.186 |
| cis-Phytol | 339 | 19.938 | 96 | 1.211 | 0.077 | 0.117 | <0.001 |
| cis-gondoic acid | 354 | 22.404 | 200 | 0.430 | 0.524 | 0.317 | 1.164 |
| ciliatine | 542 | 16.068 | 398 | 0.573 | 0.392 | 0.265 | 1.704 |
| cholesterol-2,2,3,4,4,6-d6 | 329 | 28.034 | 98 | 1.026 | 0.108 | 0.133 | 0.263 |
| cholesterol | 684 | 28.105 | 107 | 1.700 | 0.007 | 0.036 | >1000 |
| cerotinic acid | 249 | 26.843 | 299 | 1.689 | 0.002 | 0.021 | 2.029 |
| cellobiose | 814 | 24.699 | 555 | 1.697 | 0.002 | 0.020 | 3.930 |
| carnitine | 782 | 9.660 | 117 | 1.409 | 0.036 | 0.081 | >1000 |
| carbamoyl-aspartic acid | 573 | 16.653 | 257 | 1.604 | 0.014 | 0.049 | >1000 |
| canavanine degr prod | 225 | 9.523 | 111 | 0.426 | 0.528 | 0.318 | 1.768 |
| butyraldehyde | 235 | 10.513 | 150 | 0.189 | 0.782 | 0.405 | 0.901 |
| beta-Mannosylglycerate | 593 | 20.517 | 243 | 1.778 | 0.003 | 0.026 | 5.425 |
| beta-Hydroxymyristic acid | 296 | 18.866 | 297 | 1.816 | 0.000 | 0.012 | 5.467 |
| beta-Glycerophosphoric acid | 958 | 15.869 | 243 | 0.796 | 0.226 | 0.195 | 1.237 |
| beta-Glutamic acid | 919 | 14.619 | 232 | 0.494 | 0.463 | 0.294 | 1.258 |
| beta-Alanine | 947 | 12.367 | 248 | 0.753 | 0.254 | 0.209 | 0.645 |
| benzoic acid | 896 | 10.019 | 179 | 0.181 | 0.791 | 0.408 | 1.023 |
| atropine | 366 | 21.387 | 122 | 0.523 | 0.436 | 0.283 | 1.396 |
| aspartic acid | 274 | 13.430 | 188 | 1.011 | 0.114 | 0.135 | 0.650 |
| asparagine | 912 | 13.334 | 100 | 1.069 | 0.092 | 0.125 | 0.638 |
| ascorbate | 863 | 18.198 | 332 | 1.011 | 0.139 | 0.149 | 1.963 |
| arachidonic acid | 907 | 22.011 | 91 | 0.842 | 0.198 | 0.181 | 0.706 |
| arachidic acid | 704 | 22.599 | 117 | 0.863 | 0.185 | 0.175 | 0.476 |
| androsterone | 447 | 23.643 | 145 | 1.734 | 0.001 | 0.016 | 2.536 |
| aminomalonic acid | 858 | 12.882 | 218 | 1.029 | 0.107 | 0.132 | 1.972 |
| alpha-ketoisocaproic acid | 769 | 9.444 | 89 | 1.740 | 0.006 | 0.033 | <0.001 |
| alpha-ketoglutaric acid | 786 | 14.169 | 198 | 0.502 | 0.456 | 0.291 | 1.333 |
| alpha-Aminoadipic acid | 809 | 15.773 | 260 | 1.015 | 0.112 | 0.135 | 0.538 |
| allo-inositol | 410 | 17.455 | 318 | 1.208 | 0.078 | 0.117 | >1000 |
| allantoic acid | 469 | 17.535 | 188 | 0.935 | 0.148 | 0.154 | 0.290 |
| alanine | 959 | 7.900 | 116 | 0.946 | 0.143 | 0.151 | 1.273 |
| adrenaline | 269 | 17.156 | 153 | 0.635 | 0.363 | 0.252 | >1000 |
| adenosine 5-monophosphate | 970 | 27.029 | 169 | 0.897 | 0.167 | 0.165 | 1.457 |
| adenosine | 623 | 24.072 | 236 | 0.226 | 0.745 | 0.394 | 0.815 |
| aconitic Acid | 817 | 16.132 | 229 | 0.395 | 0.567 | 0.332 | 2.794 |
| 7-alpha-Hydroxycholesterol | 613 | 27.765 | 129 | 0.934 | 0.149 | 0.155 | 0.678 |
| 6-phosphogluconic acid | 706 | 22.354 | 318 | 1.154 | 0.065 | 0.109 | 1.658 |
| 6-hydroxynicotinic acid | 270 | 14.126 | 330 | 1.041 | 0.102 | 0.130 | 3.234 |
| 6-hydroxy caproic acid trimer | 75 | 26.052 | 243 | 1.141 | 0.069 | 0.111 | 0.316 |
| 5'-methylthioadenosine | 444 | 25.244 | 236 | 0.085 | 0.901 | 0.440 | 0.834 |
| 5-methoxytryptamine | 568 | 22.769 | 174 | 1.204 | 0.079 | 0.118 | >1000 |
| 5-aminovaleric acid | 733 | 14.814 | 174 | 1.618 | 0.003 | 0.029 | 10.56 |
| 5,6-dihydrouracil | 428 | 13.148 | 243 | 0.844 | 0.196 | 0.180 | 1.602 |
| 4-hydroxypyridine | 518 | 8.716 | 152 | 0.247 | 0.717 | 0.385 | 1.148 |
| 4-hydroxybutyrate | 897 | 9.781 | 147 | 1.314 | 0.030 | 0.075 | 1.833 |
| 4-hydroxybenzoic acid | 308 | 14.830 | 98 | 0.491 | 0.466 | 0.295 | 0.667 |
| 4-hydroxy-3-methoxycinnamaldehyde | 171 | 18.460 | 146 | 0.241 | 0.723 | 0.387 | 1.023 |
| 4-androsten-11beta-ol-3,17-dione | 286 | 26.383 | 127 | 1.192 | 0.055 | 0.101 | 6.206 |
| gama-aminobutyric acid | 245 | 8.660 | 100 | 1.442 | 0.014 | 0.049 | 2.432 |
| 4-acetylbutyric acid | 294 | 10.975 | 89 | 0.741 | 0.262 | 0.213 | 1.095 |
| 4-acetamidobutyric acid | 236 | 13.442 | 217 | 0.268 | 0.694 | 0.377 | 1.225 |
| 4',5-dihyrroxy-7-methoxyisoflavone | 239 | 26.260 | 221 | 1.279 | 0.036 | 0.081 | 2.388 |
| 3-Phenyllactic acid | 102 | 14.330 | 218 | 1.208 | 0.077 | 0.117 | >1000 |
| 3-phenylcatechol | 267 | 17.480 | 156 | 0.862 | 0.186 | 0.175 | 4.044 |
| 3-methylglutaric Acid | 300 | 12.235 | 172 | 1.580 | 0.005 | 0.032 | 0.607 |
| 3-methylamino-1,2-propanediol | 438 | 11.792 | 116 | 0.043 | 0.950 | 0.453 | 1.053 |
| 3-hydroxypyridine | 742 | 8.454 | 152 | 0.427 | 0.527 | 0.318 | 1.272 |
| 3-hydroxypropionic acid | 494 | 8.474 | 216 | 0.769 | 0.243 | 0.204 | 0.790 |
| 3-hydroxynorvaline | 266 | 11.565 | 144 | 0.843 | 0.197 | 0.181 | 0.553 |
| 3-hydroxy-L-proline | 321 | 12.474 | 142 | 0.837 | 0.201 | 0.182 | 0.564 |
| 3-hydroxybutyric acid | 940 | 8.703 | 147 | 1.402 | 0.018 | 0.057 | 0.545 |
| 3-cyanoalanine | 275 | 11.633 | 113 | 0.713 | 0.282 | 0.222 | 2.027 |
| 3-aminoisobutyric acid | 588 | 12.766 | 174 | 0.855 | 0.190 | 0.177 | 1.206 |
| 3,6-anhydro-D-galactose | 341 | 15.958 | 89 | 0.608 | 0.362 | 0.252 | 0.570 |
| 3,5-dihydroxyphenylglycine | 275 | 18.405 | 283 | 0.110 | 0.872 | 0.432 | 1.052 |
| 3,4-dihydroxybenzoic acid | 254 | 17.020 | 208 | 0.943 | 0.144 | 0.152 | 0.291 |
| 2-monopalmitin | 814 | 23.448 | 218 | 0.254 | 0.713 | 0.384 | 0.861 |
| 2-keto-isovaleric acid | 133 | 7.954 | 174 | 0.392 | 0.563 | 0.330 | 0.719 |
| 2-ketobutyric acid | 588 | 7.433 | 89 | 0.551 | 0.411 | 0.273 | 0.739 |
| 2-ketoadipate | 227 | 9.796 | 101 | 1.349 | 0.025 | 0.068 | 1.588 |
| 2-hydroxyvaleric acid | 684 | 9.416 | 131 | 0.667 | 0.315 | 0.235 | 0.372 |
| 2-hydroxypyridine | 860 | 6.974 | 152 | 0.812 | 0.216 | 0.190 | 1.106 |
| 2-hydroxybutanoic acid | 959 | 8.218 | 131 | 1.304 | 0.032 | 0.077 | 0.374 |
| 2-hydroxybiphenyl | 165 | 14.712 | 244 | 0.567 | 0.413 | 0.274 | 1.258 |
| 2-furoic Acid | 188 | 8.408 | 156 | 0.706 | 0.287 | 0.224 | 0.857 |
| 2-deoxyerythritol | 728 | 10.335 | 205 | 1.580 | 0.013 | 0.048 | 4.696 |
| 2'-deoxyadenosine 5'-monophosphate | 360 | 27.373 | 169 | 1.615 | 0.004 | 0.029 | 3.364 |
| 2-aminophenol | 276 | 13.823 | 221 | 0.543 | 0.418 | 0.276 | 1.591 |
| 2-amino-1-phenylethanol | 294 | 16.084 | 217 | 1.160 | 0.063 | 0.108 | 3.131 |
| 2,6-diaminopimelic acid | 285 | 17.272 | 174 | 0.818 | 0.212 | 0.188 | 0.533 |
| 2,4-diaminobutyric acid | 133 | 14.854 | 211 | 0.862 | 0.186 | 0.175 | 0.626 |
| 2,3-dihydroxypyridine | 612 | 10.879 | 240 | 0.836 | 0.201 | 0.182 | 1.265 |
| 2,2-dimethylsuccinic Acid | 169 | 10.942 | 130 | 0.411 | 0.543 | 0.324 | 0.919 |
| 1-monopalmitin | 757 | 23.709 | 371 | 0.071 | 0.917 | 0.444 | 1.039 |
| 1-methylhydantoin | 332 | 11.093 | 215 | 1.419 | 0.016 | 0.053 | 1.618 |
| 1,5-anhydroglucitol | 690 | 17.112 | 217 | 0.882 | 0.175 | 0.170 | 1.494 |
| 1,2-didecanoylglycerol | 405 | 29.937 | 155 | 1.463 | 0.021 | 0.062 | 5.768 |
| 1,2,4-benzenetriol | 371 | 14.470 | 254 | 0.101 | 0.882 | 0.435 | 1.051 |
| (-)-dihydrocarveol | 194 | 10.573 | 155 | 0.513 | 0.445 | 0.287 | 1.375 |

**Table S5** Differentially expressed metabolites among the 4 biofluids identified by one-way ANOVA and post-hoc analysis.

| **Metabolites** | **P-value** | **-log(p)** | **FDR** | **Fisher's LSD^1^** |
| --- | --- | --- | --- | --- |
| Oxoproline | ＊^2^ | 17.32 | ＊ | M-R; S-R; U-R; M-S; M-U; S-U |
| L-Threose | ＊ | 15.87 | ＊ | M-R; S-R; U-R; M-S; M-U; U-S |
| Oxalic acid | ＊ | 13.82 | ＊ | M-R; U-R; M-S; M-U; U-S |
| Glucose | ＊ | 13.55 | ＊ | R-M; R-U; S-M; M-U; S-U |
| Creatine | ＊ | 12.21 | ＊ | M-R; S-R; U-R; U-M; U-S; M-S |
| Lactose | ＊ | 10.26 | ＊ | M-R; R-S; U-R; M-S; U-M; U-S |
| Alanine | ＊ | 9.76 | ＊ | R-M; U-R; S-M; U-M |
| Lyxose | ＊ | 9.15 | ＊ | R-S; R-U; M-S; M-U; S-U |
| Phosphate | ＊ | 8.91 | ＊ | M-R; U-R; M-S; U-S |
| Thymol | ＊ | 8.78 | ＊ | R-M; S-M; U-M |
| Phenylethylamine | ＊ | 7.64 | ＊ | R-M; S-M; U-M |
| L-Malic acid | ＊ | 6.1 | ＊ | M-R; R-U; M-S; M-U; S-U |
| 2,4-diaminobutyric acid | ＊ | 5.6 | ＊ | R-M; S-M; U-M |
| Hydroxylamine | ＊ | 5.21 | ＊ | R-M; R-S; U-M; U-S |
| Noradrenaline | ＊ | 4.33 | ＊ | R-M; S-M; U-M |
| M-cresol | ＊ | 4.04 | ＊ | U-R; U-M; U-S |
| 5-Methoxytryptamine | ＊ | 3.51 | ＊ | R-M; R-S; U-M; U-S |
| Glycerol | ＊ | 3.42 | ＊ | R-S; R-U; M-U; S-U |
| Glycine | ＊ | 2.94 | ＊ | R-M; S-M; U-M |
| Fumaric acid | ＊ | 2.14 | 0.01 | R-U; S-U |
| Aminomalonic acid | 0.011 | 1.98 | 0.015 | S-M |
| N-Methyl-L-glutamic acid | 0.012 | 1.93 | 0.016 | R-S; U-S |
| Aminomalonic acid | 0.021 | 1.68 | 0.026 | S-R; S-M |
| Lactic acid | 0.023 | 1.63 | 0.028 | S-U |
| Malonic acid | 0.043 | 1.37 | 0.05 | R-M; U-M |
| 2-hydroxybutanoic acid | 0.048 | 1.32 | 0.054 | S-R; U-R |

^1^LSD = least significant difference. R=rumen fluid; S=serum; M=milk; U=urine. M-R indicates that the comparison between milk and rumen fluid was significantly different (P < 0.05)
^2^＊value <0.01. Statistical significance was defined at P< 0.05, with high significance defined at P < 0.01. FDR < 0.05 indicated statistical significance after multiple comparisons test, with high significance at FDR < 0.01.

**Table S6** Significantly up-regulated pathways in lactation group

| ID | Pathway name |
| --- | --- |
| 1 | Citrate cycle (TCA cycle) |
| 2 | Glyoxylate and dicarboxylate metabolism |
| 3 | Glutamine metabolism |
| 4 | Glycine biosythesis and degradation |
| 5 | Butanoate metabolism |
| 6 | Arginine and proline metabolism |
| 7 | Nitrogen metabolism |
| 8 | Purine metabolism |
| 9 | Glutathione metabolism |
| 10 | D-Glutamine and D-glutamate metabolism |
| 11 | Cyanoamino acid metabolism |
| 12 | Glycine, serine and threonine metabolism |
| 13 | Steroid hormone biosynthesis |
| 14 | Methane metabolism |
| 15 | Pentose and glucuronate interconversions |
| 16 | Pentose phosphate pathway |
| 17 | Propanoate metabolism |
| 18 | Lysine degradation |
| 19 | Pyruvate metabolism |
| 20 | Porphyrin and chlorophyll metabolism |
| 21 | Cysteine and methionine metabolism |
| 22 | Pyrimidine metabolism |
